# Facile synthesis of carbon nanotube-supported NiO//Fe_2_O_3_ for all-solid-state supercapacitor*s*

**DOI:** 10.3762/bjnano.10.188

**Published:** 2019-09-23

**Authors:** Shengming Zhang, Xuhui Wang, Yan Li, Xuemei Mu, Yaxiong Zhang, Jingwei Du, Guo Liu, Xiaohui Hua, Yingzhuo Sheng, Erqing Xie, Zhenxing Zhang

**Affiliations:** 1 Key Laboratory for Magnetism and Magnetic Materials of the Ministry of Education, Key Laboratory of Special Function Materials and Structure Design of the Ministry of Education, School of Physical Science and Technology, Lanzhou University, Lanzhou 730000, China

**Keywords:** aqueous reduction, carbon nanotubes, iron oxide, nickel oxide, supercapacitors

## Abstract

We have successfully prepared iron oxide and nickel oxide on carbon nanotubes on carbon cloth for the use in supercapacitors via a simple aqueous reduction method. The obtained carbon cloth–carbon nanotube@metal oxide (CC-CNT@MO) three-dimensional structures combine the high specific capacitance and rich redox sites of metal oxides with the large specific area and high electrical conductivity of carbon nanotubes. The prepared CC-CNT@Fe_2_O_3_ anode reaches a high capacity of 226 mAh·g^−1^ at 2 A·g^−1^ with a capacitance retention of 40% at 40 A·g^−1^. The obtained CC-CNT@NiO cathode exhibits a high capacity of 527 mAh·g^−1^ at 2 A·g^−1^ and an excellent rate capability with a capacitance retention of 78% even at 40 A·g^−1^. The all-solid-state asymmetric supercapacitor fabricated with these two electrodes delivers a high energy density of 63.3 Wh·kg^−1^ at 1.6 kW·kg^−1^ and retains 83% of its initial capacitance after 5000 cycles. These results demonstrate that our simple aqueous reduction method to combine CNT and metal oxides reveals an exciting future in constructing high-performance supercapacitors.

## Introduction

Supercapacitors offer long cycling life, superior charge–recharge ability, high power density, and wide operating temperature [1–3]. However, the low energy density limits their application in various energy-consuming devices. Many materials have been explored to be used in supercapacitors to increase their energy density [4–5]. Carbon materials, especially carbon nanotubes and graphene, endowed with good conductivity and high specific surface area, are ideal candidates, and they are widely used in commercial supercapacitors [6–9]. Although they have a higher capacity than the conventional capacitors, their average energy density is low to about 10 Wh·kg^−1^ whereas batteries reach 200 Wh·kg^−1^. Transition metal oxides such as RuO_2_, MnO_2_, NiO, and Fe_2_O_3_ [10–15] have a high theoretical capacity from faradaic reactions but suffer from low conductivity, small surface area, and poor cycling stability, resulting in low capacitance and poor rate capability [16]. To address these issues, many researchers have devoted enormous efforts to combine carbon materials with metal oxides [17]. Among them, carbon nanotubes combined with Fe_2_O_3_ have attracted considerable attention. Fe_2_O_3_ is attractive for its low cost, abundance, nontoxicity, and eco-friendliness [18–20]. Some great results on CNT@Fe_2_O_3_ composites have been achieved. For example, Guan et al. deposited iron oxide on CNTs by atomic layer deposition (ALD) and the obtained CNTs@Fe_2_O_3_ presented a specific capacitance of 580.6 F·g^−1^ at 5 A·g^−1^ [21]. Zhang et al. used magnetron sputtering to prepare sandwich-like CNT@Fe_2_O_3_@C structures, and the composite exhibited a specific capacitance of 787.5 F·g^−1^ at 5 mV·s^−1^ [19]. These methods have successfully improved the performance of the electroactive material, but the expensive instruments or complex synthesis processes have hindered a broad application.

Considering all the above issues, we proposed a simple solution-processing method to synthesize Fe_2_O_3_ on a carbon cloth–carbon nanotubes (CC-CNT) substrate with NaBH_4_ as reductant and ferric chloride as the reactant. NaBH_4_ is an active reducing agent with which most metal chlorides can be reduced to metal [22–24] and then be oxidized in air to form CNT@metal oxide composites. We also adapted this method to prepare CNT@NiO composites. NiO is a potential positive material offering high theoretical capacity, nontoxicity, and environmentally benign nature [25].

Through aqueous reduction, Fe_2_O_3_-coated CNT on carbon cloth (CC-CNT@Fe_2_O_3_) as anode and NiO-coated CNT on carbon cloth (CC-CNT@NiO) as cathode were prepared. CNTs significantly improved the conductivity and enhanced the capacity of Fe_2_O_3_ up to 226 mAh·g^−1^ at 2 A·g^−1^, and capacity of NiO to 527 mAh·g^−1^ at 2 A·g^−1^. Furthermore, by assembling the two electrodes, an asymmetric supercapacitor (ASC) with a high energy density of 63.3 Wh·kg^−1^ at 1.6 kW·kg^−1^ was obtained. Besides, the ASC exhibits excellent cycling stability with capacitance retention of 83% after 5000 cycles. All these results demonstrate that our method is promising for constructing CNT-based metal oxide composites for high-performance supercapacitors.

## Results and Discussion

**Figure 1:**
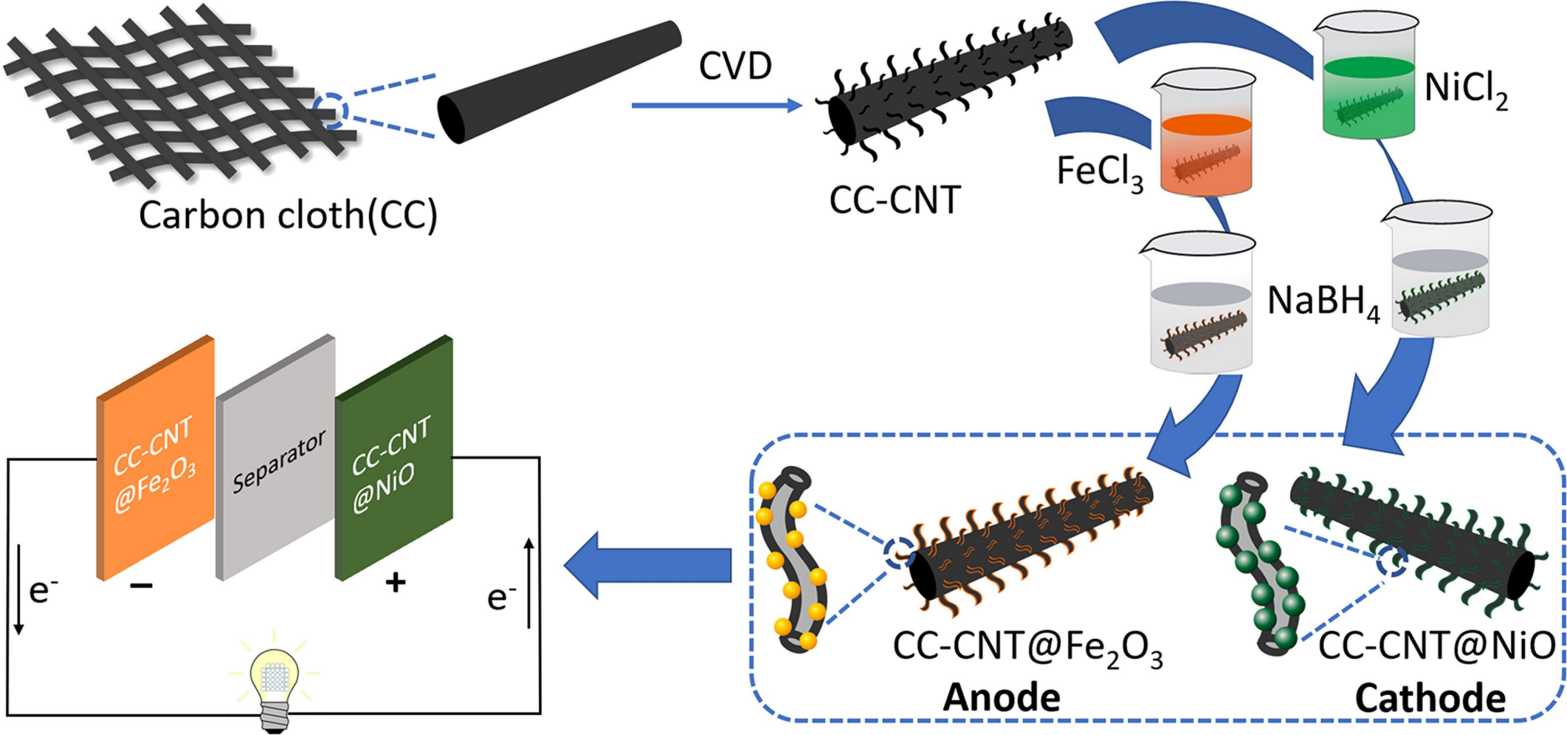
Schematic illustration of the preparation of the asymmetric supercapacitor.

Figure 1 shows the process of synthesizing cathode and anode, and finally, the asymmetric supercapacitor. The details can be seen in the Experimental section.

### Anode material CC-CNT@Fe_2_O_3_

**Figure 2:**
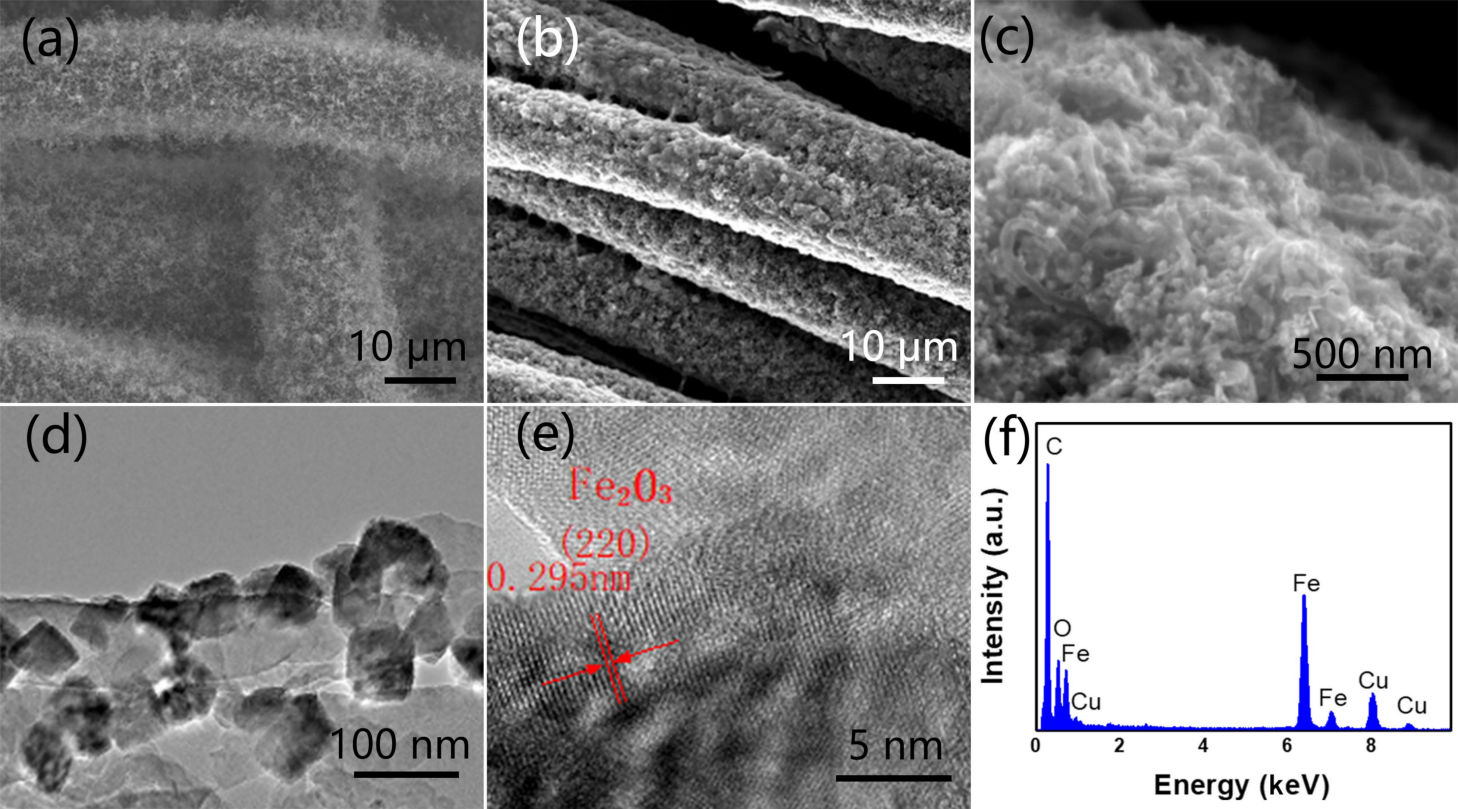
SEM images of (a) CNTs grown on carbon cloth; (b) CC-CNT@Fe_2_O_3_; (c) magnified CC-CNT@Fe_2_O_3_. (d) TEM image of Fe_2_O_3_ particles with a diameter of 50 nm on CNT. (e) High-resolution TEM image of Fe_2_O_3_ with an interplanar spacing of 0.295 nm, corresponding to the (220) plane of Fe_2_O_3_. (f) EDX spectrum of the CC-CNT@Fe_2_O_3_.

CNTs were grown on CC by chemical vapour deposition (CVD). As shown in Figure 2a, CNTs grow homogeneously with a diameter of about 20 nm (Supporting Information File 1, Figure S1). The high conductivity of the CNTs will help charge transfer during the electrochemical process. As shown in Supporting Information File 1, Figure S2, the pure CC with a size of 1 × 1 cm^2^ is grey, it becomes black after CNT growth and finally yellow after Fe_2_O_3_ growth. The mass loading of Fe_2_O_3_ can be adjusted through the concentration of FeCl_3_. As shown in Supporting Information File 1, Figure S3a, the mass loading increases linearly as the concentration increases, implying the controllable growth of Fe_2_O_3_. The morphology of the as-obtained CC-CNT@Fe_2_O_3_ is shown in Figure 2b, where Fe_2_O_3_ coats all carbon fibers well. The space between CNTs provides enormous room for Fe_2_O_3_ growth, and the obtained CNT@Fe_2_O_3_ are entangled with each other and sticky to the CC (Figure 2c). N_2_ adsorption–desorption isotherm and Barrett–Joyner–Halenda (BJH) pore size distribution of CC-CNT@Fe_2_O_3_ are shown in Supporting Information File 1, Figure S4. It is a type-IV isotherm, indicating the mesoporous texture of the sample (Figure S4a, Supporting Information File 1). The BET surface area was found to be 24.9 m^2^·g^−1^, much larger than that of pure carbon cloth (0.2 m^2^·g^−1^). The pore size distribution (Figure S4b, Supporting Information File 1) shows that most pores have a size of 40–50 nm. The high surface area, and the mesopores can help the ion diffusion between electrode and electrolyte. The morphologies of CNT@Fe_2_O_3_ were further examined by TEM. As shown in Figure 2d, Fe_2_O_3_ nanoparticles with a size of about 50 nm are distributed evenly on the CNTs. The high-resolution TEM image (Figure 2e) indicates an interplanar spacing of 0.295 nm, corresponding to the (220) plane of Fe_2_O_3_ (JCPDS Card No. 25-1402). The EDX spectrum confirms the existence of C, O, and Fe elements (Figure 2f, Cu signal is due to the copper grid).

**Figure 3:**
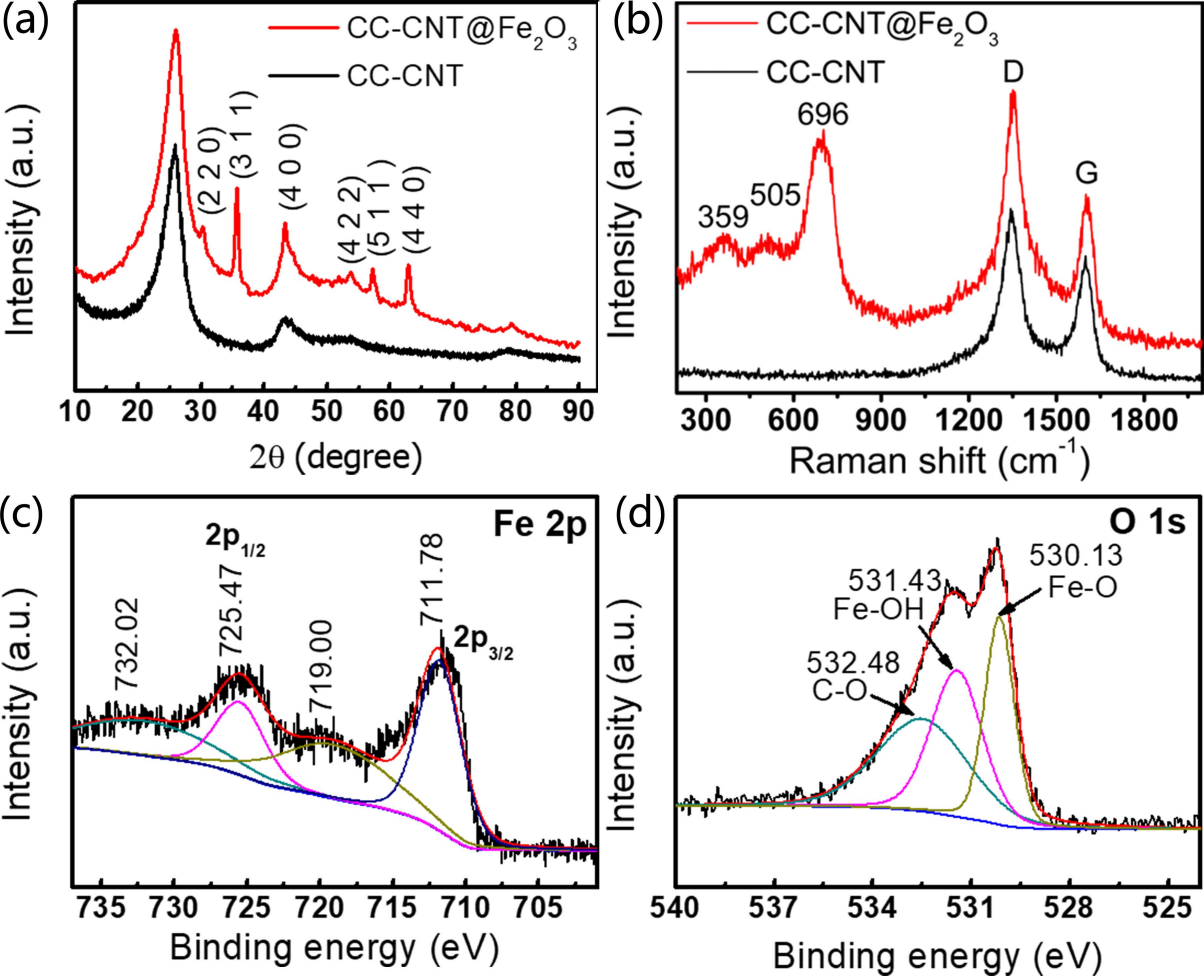
(a) XRD pattern and (b) Raman spectra of CC-CNT and CC-CNT@Fe_2_O_3_; (c) Fe 2p and (d) O 1s XPS spectra of CC-CNT@Fe_2_O_3_.

The crystallographic structures of the samples are shown in Figure 3a. Excluding the peaks of CC-CNT, all other diffraction peaks can be assigned to Fe_2_O_3_ (JCPDS Card No. 25-1402). The samples show the same XRD features at different mass loadings (Supporting Information File 1, Figure S3b), indicating the consistent synthesis of Fe_2_O_3_. In addition, no other diffraction peaks of impurities are observed, demonstrating the successful synthesis of pure Fe_2_O_3_. Furthermore, Raman spectra (Figure 3b) shows three peaks at 359, 505, and 696 cm^−1^, corresponding to T_2g_, E_g_, and A_1g_ modes of Fe_2_O_3_, verifying the existence of Fe_2_O_3_ in CC-CNT@Fe_2_O_3_ [26]. The G-band at 1604 cm^−1^ and the D-band at 1344 cm^−1^ originate from sp^2^-hybridized carbon atoms and imperfections in CNTs, respectively [27]. It should be noted that the broad peaks in both XRD pattern and Raman spectra indicate that Fe_2_O_3_ is not well crystallized since it was formed at 70 °C in the drying oven without further annealing. The XPS spectrum in Figure S5a (Supporting Information File 1) shows the existence of Fe, O, and C elements in CC-CNT@Fe_2_O_3_. The Fe 2p spectrum (Figure 3c) shows 2p_1/2_ and 2p_3/2_ at 725.47 eV and 711.78 eV, respectively, with the doublet separation of 13.69 eV. There are two typical satellite peaks at 719.00 eV and 732.02 eV, indicating the Fe^3+^ oxidation state [28–29]. The O 1s spectrum (Figure 3d) shows three peaks at 532.48 eV, 531.43 eV, and 530.13 eV, corresponding to C–O, Fe–O–C, and Fe–O, respectively [30]. The XPS results strongly support the XRD and Raman results and confirm Fe_2_O_3_ on the CC-CNT.

**Figure 4:**
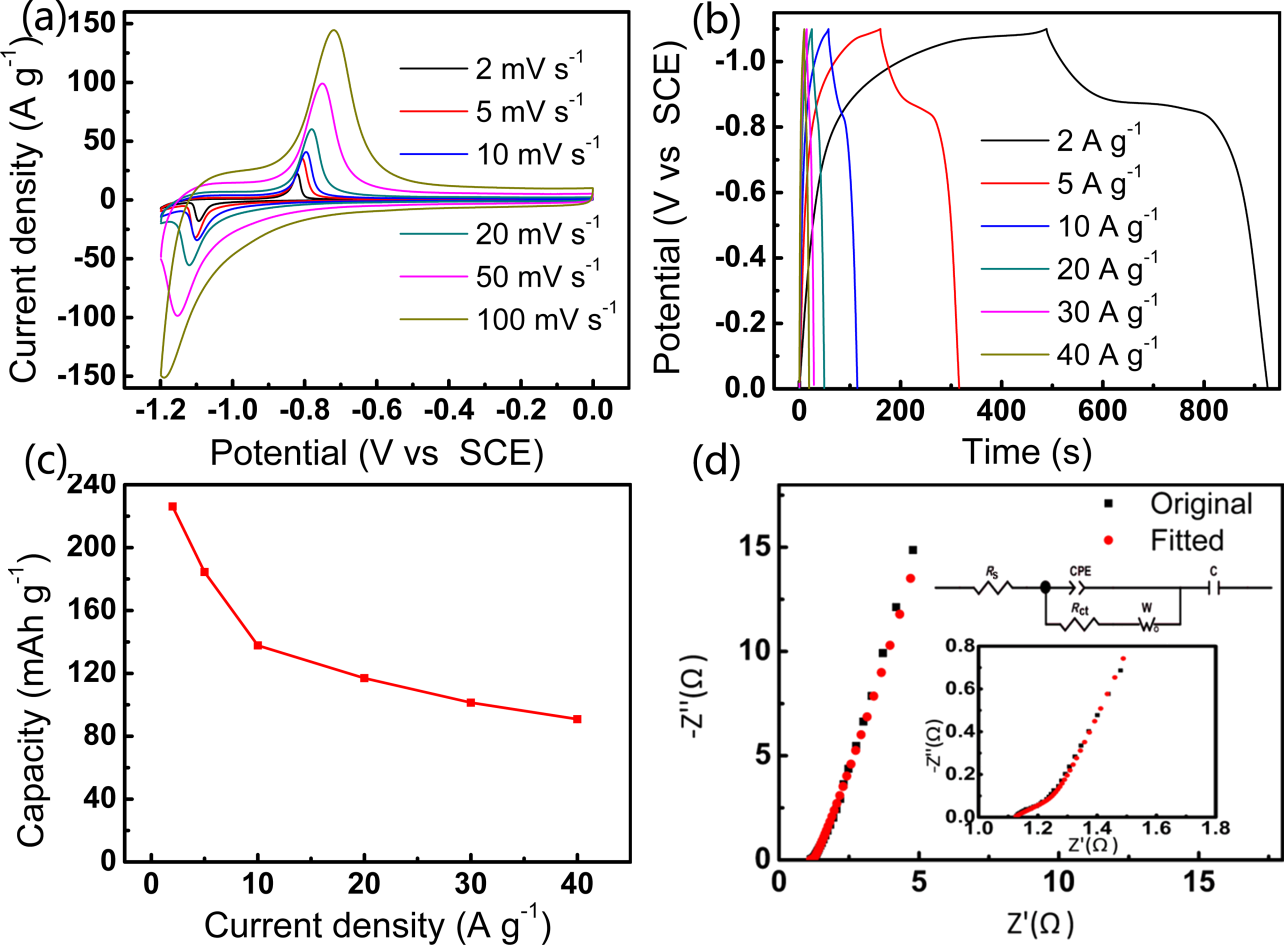
Electrochemical performance of the CC-CNT@Fe_2_O_3_ electrode: (a) CV curves; (b) GCD curves; (c) capacity versus current density; (d) EIS curves.

A three-electrode system was used to examine the electrochemical characteristics of the CC-CNT@Fe_2_O_3_ with Pt foil as a counter electrode, SCE as a reference electrode, CC-CNT@Fe_2_O_3_ as the binder-free working electrode and 2 M KOH as the electrolyte. Figure 4a shows cyclic voltammetry (CV) curves of the CC-CNT@Fe_2_O_3_ electrode at scan rates of 2, 5, 10, 20, 50 and 100 mV·s^−1^, from which visible redox peaks can be seen. The reduction peak at ca. −1.1 V and the oxidation peak at ca. −0.7 V can be attributed to the reaction between Fe^3+^ and K^+^ in the electrolyte [21,31]. When plotting log *i* versus log *v* of the redox peaks according to the empirical Randles–Sevcik equation [32–33],

[1] logi(v)=loga+blogv,

the slope can be determined to be about 0.58, indicating a battery-like behaviour of Fe_2_O_3_ [34]. We also compared CC-CNT@Fe_2_O_3_ with CC-CNT. As shown in Supporting Information File 1, Figure S6a, the CV area of CC-CNT is much smaller than that of CC-CNT@Fe_2_O_3_, indicating that Fe_2_O_3_ plays a predominant role in the electrode. The galvanostatic charge/discharge (GCD) test yields the same result (Figure S6b, Supporting Information File 1), where the charge–discharge time of CC-CNT is much shorter than that of CC-CNT@Fe_2_O_3_. GCD curves of the CC-CNT@Fe_2_O_3_ electrode at different densities from 2 to 40 A·g^−1^ are presented in Figure 4b. A clear charge–discharge plateau can be seen, demonstrating the battery-like behaviour of the electrode. The capacities are 226, 184, 137, 116, 101, and 91 mAh·g^−1^ (based on the mass of Fe_2_O_3_, 0.43 mg) at current densities of 2, 5, 10, 20, 30, and 40 A·g^−1^, respectively (Figure 4c), indicating excellent rate capability of the CC-CNT@Fe_2_O_3_ electrode. Electrochemical impedance spectroscopy (EIS) was carried out to explore the resistance of the electrode (Figure 4d). The measured curve can be well fitted by equivalent circuits, and the insert is an enlarged view in the high-frequency area. All fitted values are shown in Supporting Information File 1, Table S1. *R*_s_ and *R*_ct_ are calculated to be 1.125 Ω and 0.002 Ω, respectively, indicating a high conductivity of the electrode. This good conductivity will contribute a lot to the electrochemical performance of the electrode. Besides, the high slope value at low frequencies indicates low ion diffusion resistance of the electrode (0.8 Ω). Therefore, the EIS results confirm the good conductivity of CC-CNT@Fe_2_O_3_ 3D structures, which contributes much to the superior electrochemical performance of the CC-CNT@Fe_2_O_3_ anode.

### Cathode material CC-CNT@NiO

**Figure 5:**
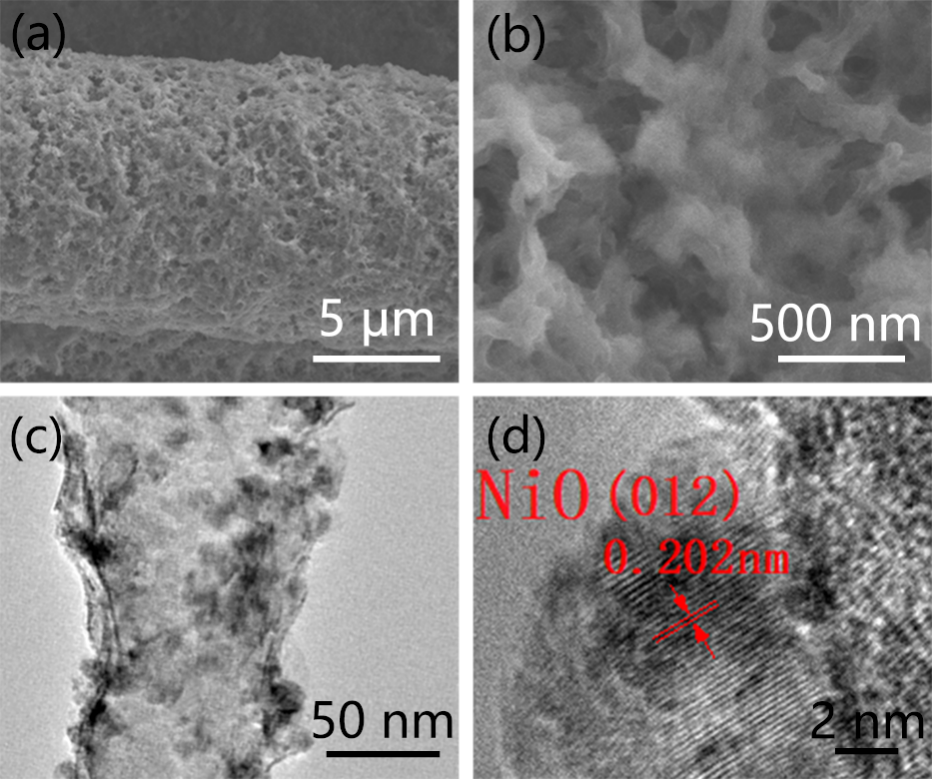
SEM images of (a) CC-CNT@NiO; (b) magnified CC-CNT@NiO. (c) TEM image of NiO particles on CNT; (d) high-resolution TEM image of NiO.

**Figure 6:**
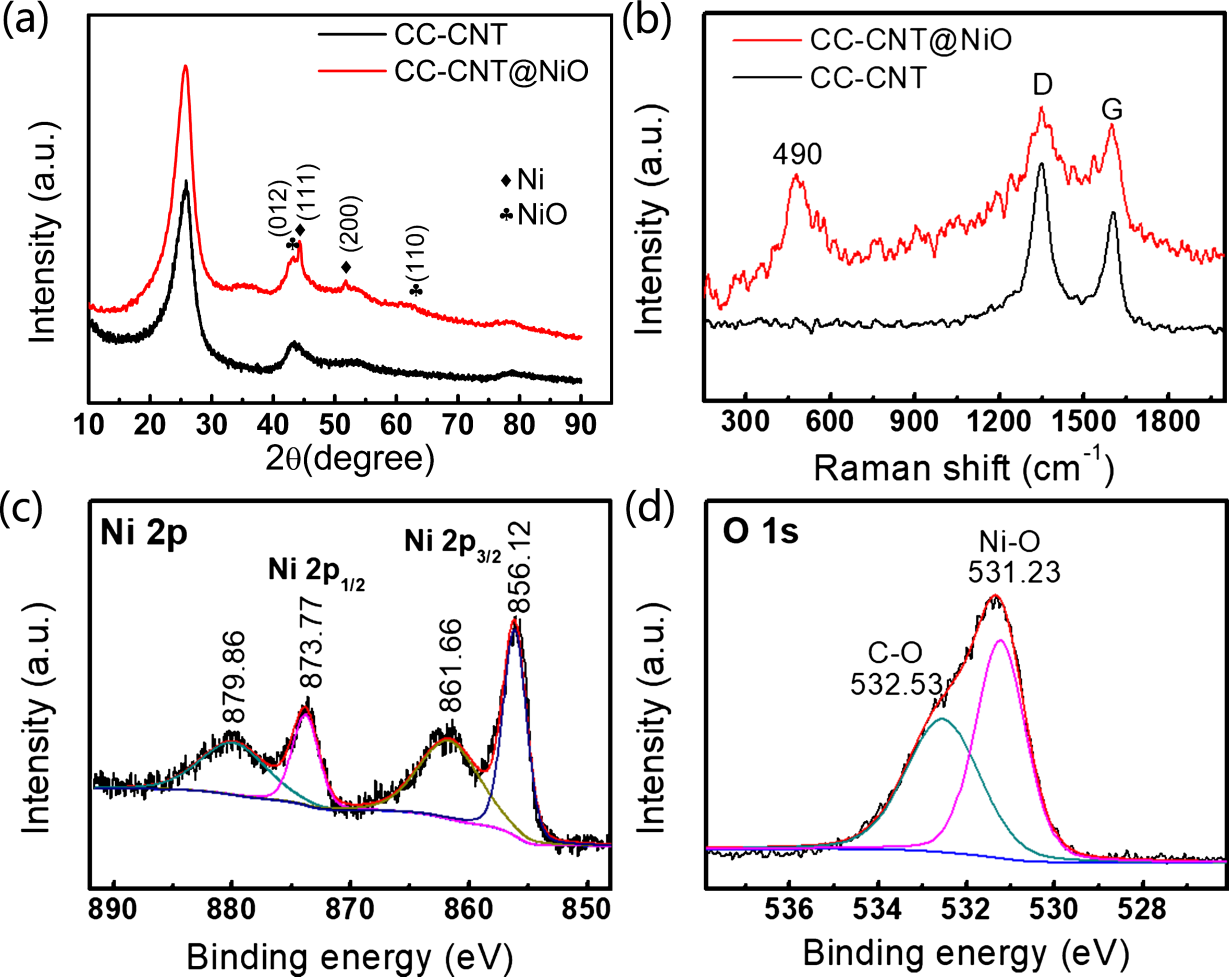
Characterization of CC-CNT@NiO: (a) XRD; (b) Raman; (c) Ni 2p and (d) O 1s XPS spectra.

NiO was successively grown on the CC-CNT substrate by the same method as Fe_2_O_3_. As shown in Figure 5a, NiO is homogeneously coated on CC-CNT forming porous structures. N_2_ adsorption–desorption isotherm and BJH pore size distribution of CC-CNT@NiO are shown in Supporting Information File 1, Figure S7. It is a type-IV isotherm, verifying the mesoporous texture of the sample (Figure S7a, Supporting Information File 1). The BET surface area was 34.4 m^2^·g^−1^, much larger than that of pure carbon cloth (0.2 m^2^·g^−1^). Pore size distribution (Figure S4b, Supporting Information File 1) shows that numerous pores have a size of about 2 and 50 nm. The high surface area and the mesopores can speed up the ion diffusion between electrode and electrolyte. As can be seen from Figure 5b, the NiO-coated CNTs are entangled with each other. These interconnected structures not only increase the surface area of the electrode to facilitate fast ion transport but also help the electrons transfer because of the excellent conductivity of CNTs. In the detailed image of CC-CNT@NiO shown in Figure 5c, the NiO nanoparticles adhered to CNT can be seen. The HR-TEM image in Figure 5d yields an interplanar spacing of 0.202 nm corresponding to the (012) plane of NiO (JCPDS Card No. 44-1159). Besides, the EDX spectrum indicates the existence of only Ni, O, and C elements in the electrode material (Figure S8, Supporting Information File 1). Furthermore, XRD and Raman confirmed NiO in the sample. The XRD pattern (Figure 6a) shows two peaks at 43.2° and 62.9°, which can be ascribed to (012) and (110) planes of NiO (JCPDS Card No. 44-1159). Apart from NiO, we can also see the peaks at 44.5° and 51.8°, responding to (111) and (200) planes of Ni (JCPDS Card No. 04-0850). This result reveals that NiO has a Ni core. The Raman spectrum shows an additional peak at 490 cm^−1^ of CC-CNT@NiO, when compared to the pure CC-CNT substrate, verifying successful synthesis of NiO on the CC-CNT substrate (Figure 6b) [35]. The XPS spectrum in Figure S5b (Supporting Information File 1) also demonstrates the existence of Ni, O, and C in CC-CNT@NiO. The spectrum of Ni 2p (Figure 6c) shows the Ni 2p_1/2_ and Ni 2p_3/2_ at 873.77 eV and 856.12 eV, respectively, along with two satellite peaks at 879.88 eV and 861.66 eV, indicating the existence of Ni in the oxidation state Ni^2+^ [12,36]. The spectrum of O 1s (Figure 6d) shows two peaks at 532.53 eV and 531.23 eV, corresponding to C–O and Ni–O, respectively [36]. The XPS results strongly support XRD and Raman results and confirm that NiO formed on the CC-CNT substrate, suggesting the universality of this aqueous reduction method to prepare CNT-based metal oxide composite.

**Figure 7:**
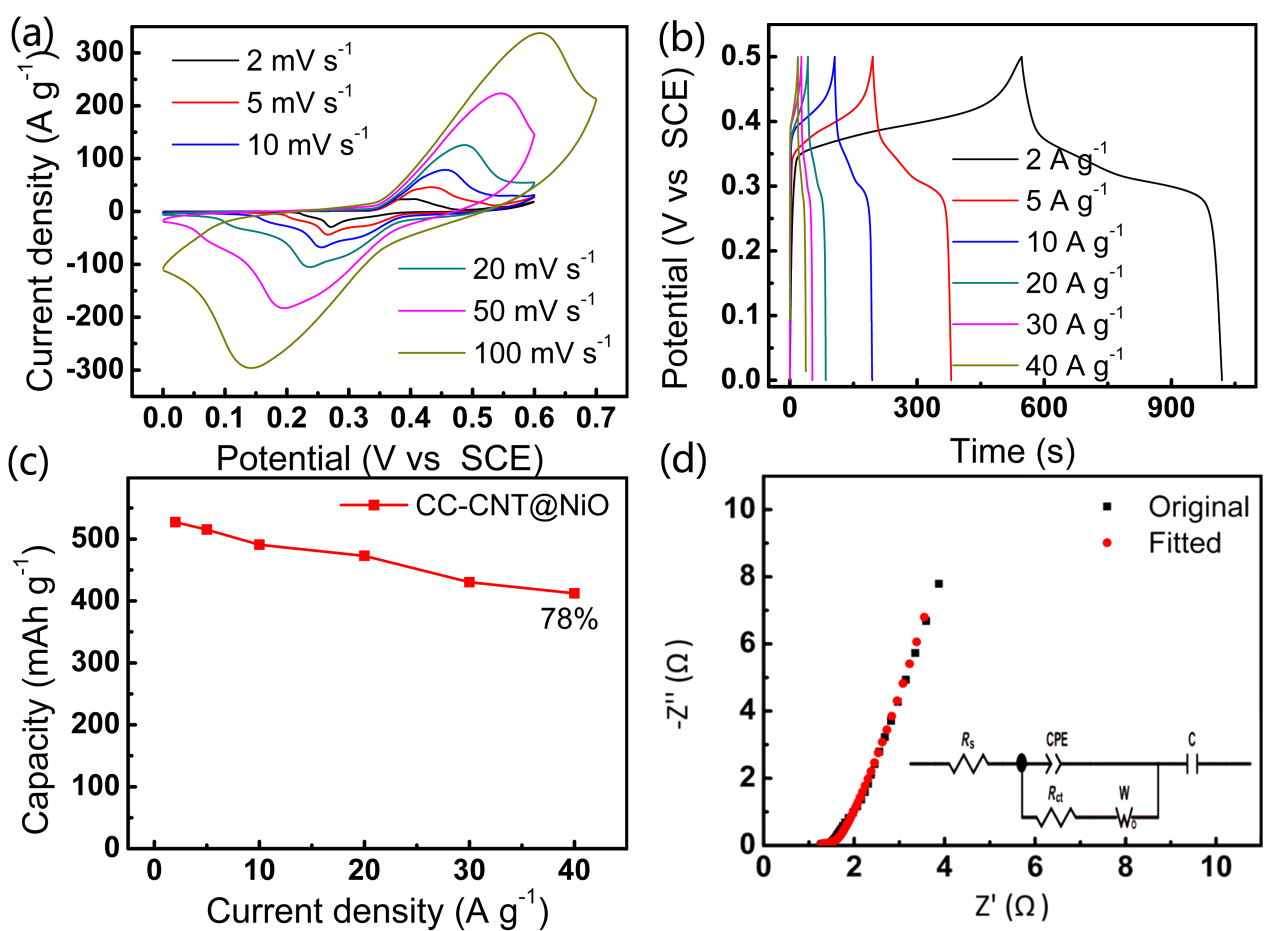
Electrochemical performance of the CC-CNT@NiO electrode: (a) CV curves; (b) GCD curves; (c) capacity versus current density; (d) EIS curve.

The electrochemical performance of the CC-CNT@NiO electrode was examined. Figure 7a shows the CV curves at 2, 5, 10, 20, 50 and 100 mV·s^−1^, from which obvious redox peaks between Ni^2+^ and OH^−^ can be seen, the redox equation is [37]:

[2] $$\text{NiO}+{\text{OH}}^{-}↔\text{NiOOH}+{\text{e}}^{-}.$$

By plotting log *i* versus log *v* of the redox peaks according to Equation 1, the slope is determined to be about 0.7, indicating the surface and diffusion-controlled capacitance both play an essential role in the total capacitance. We also compared CC-CNT@NiO with CC-CNT. As shown in Supporting Information File 1, Figure S9a, the CV integrated area of CC-CNT is smaller than CC-CNT@NiO, indicating that NiO plays a predominant role in the electrode. The galvanostatic charge/discharge (GCD) test yields the same result (Figure S9b, Supporting Information File 1), where the charge–discharge time of CC-CNT is negligible compared to that of CC-CNT@NiO. GCD curves at different densities from 2 to 40 A·g^−1^ are presented in Figure 7b. The calculated capacities are 527, 515, 491, 473, 430 and 412 mAh·g^−1^ (based on the mass of NiO, 0.33 mg) at current densities of 2, 5, 10, 20, 30, and 40 A·g^−1^, respectively (Figure 7c). At the large current density of 40 A·g^−1^ still 78% of the highest capacity of 527 mAh·g^−1^ at 2 A·g^−1^ is retained, indicating excellent rate capability of the CC-CNT@NiO electrode. Moreover, as shown in the EIS curve of Figure 7d, the equivalent serial resistance is 1.27 Ω (all the fitted values are shown in Supporting Information File 1, Table S1), indicating the high conductivity of the electrode. Also, the high slope at low frequency indicates small capacitive resistance (0.465 Ω) and thus fast ion transport. Therefore, the EIS results prove that the good electrochemical performance of the CC-CNT@NiO electrode can be mainly attributed to its good electrical conductivity and low charge transfer resistance.

### All-solid-state asymmetric supercapacitor

**Figure 8:**
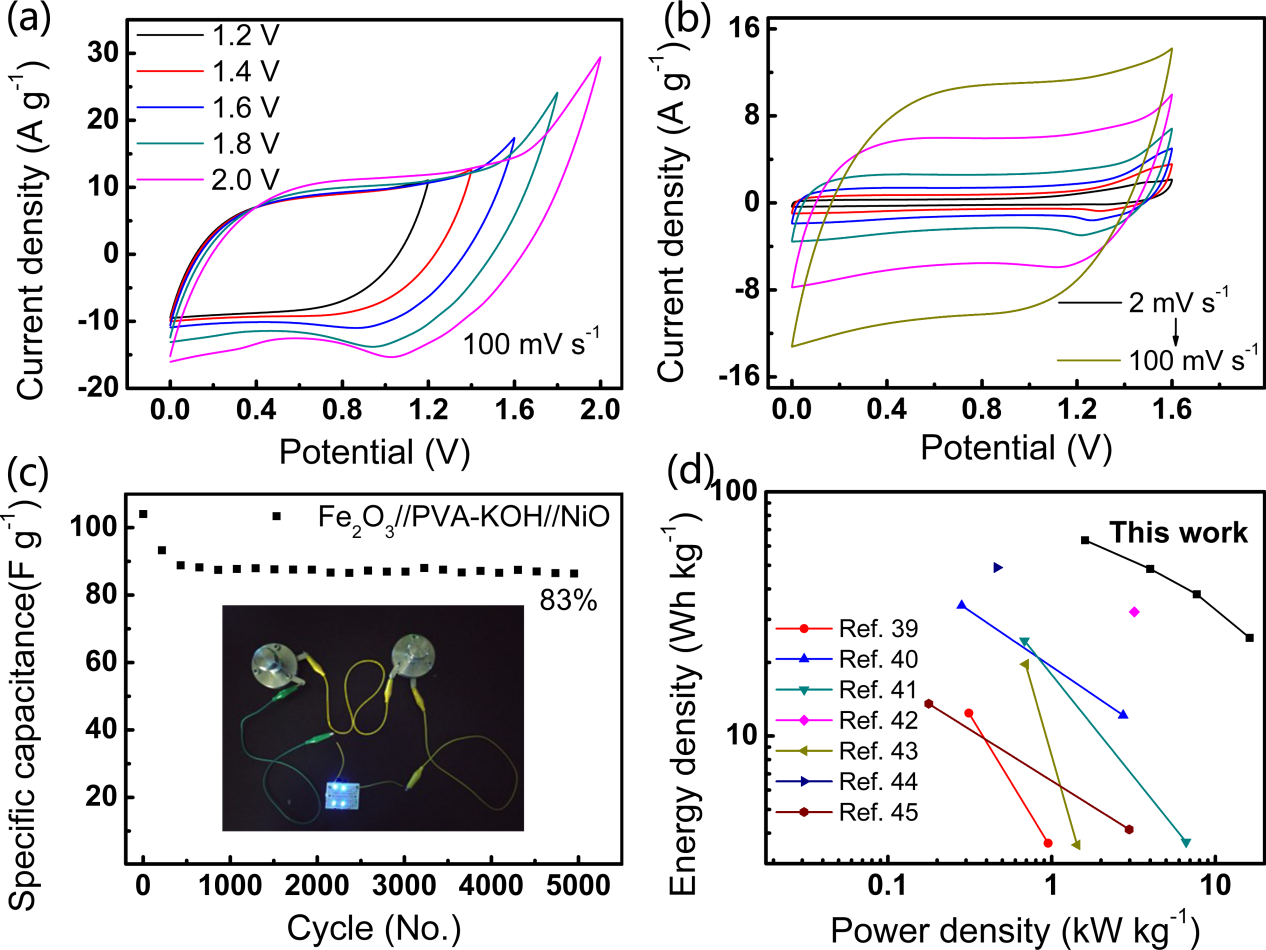
(a) CV curves of the all-solid-state ASC recorded at voltage windows from 1.2 to 2.0 V. (b) CV curves of the device performed at scan rates from 2 to 100 mV·s^−1^ at 1.6 V. (c) Cycling test of the device with 83% retention after 5000 cycles. The insert shows the demo of two devices in series which can power four blue LEDs. (d) Ragone plot of the device compared with previously reported results [39–45].

An all-solid-state asymmetric supercapacitor (ASC) was constructed with CC-CNT@Fe_2_O_3_ as the negative electrode, CC-CNT@NiO as the positive electrode, potassium hydroxide/polyvinyl alcohol gel (KOH/PVA, 1:1 mass ratio) as the electrolyte, and a cellulose membrane (140 μm thick) as the separator. To assemble the device with the best performance, charge balance between the positive and negative electrodes were considered by adjusting the mass loading of the active materials and verified by the areal ratio of CV at the same scan rate of 20 mV·s^−1^ (Supporting Information File 1, Figure S10a). Once the charge is balanced (0.15 mg Fe_2_O_3_ vs 0.23 mg NiO), CV curves of the ASC at different voltage windows were measured to find out the optimal operating voltage window. As shown in Figure 8a, there is severe polarization when the window is larger than 1.6 V. As a result, the optimal voltage window is chosen to be 1.6 V in subsequent tests. CV curves of the ASC at different scan rates are presented in Figure 8b. The CV curves are quasi-rectangular, revealing that the ASC displays a superior electrochemical performance, which mainly originates from the deliberately designed 3D structures of both the CC-CNT@Fe_2_O_3_ and CC-CNT@NiO electrodes. When the scan rate increases from 2 to 100 mV·s^−1^, the CV shapes are still maintained, suggesting the ultrafast charge–discharge ability and excellent reversibility of the device. The GCD curves of the device measured at 2, 5, 10 and 20 A·g^−1^ are shown in Figure S10b (Supporting Information File 1). An obvious IR drop can be seen in each curve. The IR drop is the electrical potential difference between the two ends of a conducting phase during a current flow. It is determined by the resistance of the device and the experimental current. Since the conductivity of PVA-KOH is not as high as the aqueous electrolyte, the resistance could be rather high. IR drop is 0.038 V at 2 A·g^−1^, and due to that it intends to increase with current, thus is pretty high at 20 A·g^−1^ [38]. The specific capacitance is calculated to be 178, 136, 107, and 71 F·g^−1^ at current densities of 2, 5, 10, and 20 A·g^−1^ (Figure S10c, based on the mass of Fe_2_O_3_ and NiO), respectively. Cycling stability of the device was conducted at 10 A·g^−1^ for 5000 cycles. As shown in Figure 8c, 83% of the initial capacitance is retained, demonstrating the high stability of the device. Also, the EIS curves in Figure S10d reveal that the charge transfer resistance of the device increases after cycling, which is responsible for the capacity decay after cycling. Besides, the insert shows the demo of the two devices in series, which can power four blue LEDs (3.0 V for each LED) in parallel, indicating good practicability of the device. In addition, it can continuously power a blue LED for nearly 27 s (video in Supporting Information File 2). The Ragone plot of the device is presented in Figure 8d with some previously reported results. The device exhibits a high energy density of 63.3 Wh·kg^−1^ at 1.6 kW·kg^−1^ and retains 25.2 Wh·kg^−1^ at a high power density of 16.2 kW·kg^−1^. These values are superior to the previously reported NiO-based or Fe_2_O_3_-based solid-state ASCs [39–45]. These impressive results originate from the remarkable performance of both positive CC-CNT@NiO and negative CC-CNT@Fe_2_O_3_ electrodes. Therefore, the rationally designed CNT@metal oxide 3D structures made by the aqueous reduction method are promising for constructing high-performance supercapacitors.

## Conclusion

Fe_2_O_3_ and NiO were individually synthesized on carbon nanotubes on carbon cloth by a simple aqueous reduction method. The prepared negative CC-CNT@Fe_2_O_3_ and positive CC-CNT@NiO electrodes both show high capacity, excellent rate capability, and high conductivity. Additionally, an all-solid-state asymmetric supercapacitor with CC-CNT@NiO as the positive electrode, CC-CNT@Fe_2_O_3_ as the negative electrode, and PVA/KOH gel as the electrolyte delivered a high energy density of 63.3 Wh·kg^−1^ at 1.6 kW·kg^−1^. Moreover, the device exhibits superior cycling stability with 83% retention of initial capacitance after 5000 cycles. The outstanding electrochemical performance of the device stems from the rationally designed CNT@metal oxide 3D structures. More importantly, our simple method provides a new strategy for constructing CNT@metal oxide composites for high-performance supercapacitors.

## Experimental

All the reagents were of analytical grade and used without further purification. The preparation of the positive and negative electrodes and the device structures are illustrated in Figure 1. The detailed process is as follows:

### Fabrication of carbon nanotubes on carbon cloth (CC-CNT)

Carbon nanotubes (CNTs) were grown on carbon cloth (CC) (CeTech Co., Ltd.) by CVD with a pretreatment of the CC. Typically, the CC (1 × 1 cm^2^, 11 mg) was treated with a mixture of H_2_SO_4_/HNO_3_ (3:1 volume ratio) at 70 °C for 2 h and then cleaned by sonication in deionized water and finally kept in a drying oven for 12 h. Afterward, the CC was immersed in catalyst, which consists of 10 mM Ni(NO_3_)_2_ and 1 mM Al(NO_3_)_3_ in alcoholic solution before CVD treatment. Flow rates of C_2_H_2_, H_2_, and Ar were set to 10, 20, and 50 sccm. The growth time was 30 min at 700 °C, and the mass loading of CNTs was 1.30 to 1.70 mg·cm^−2^. The as-prepared CNTs on CC (CC-CNT) was subsequently treated by oxygen plasma to make it hydrophilic and then immersed in diluted hydrochloric acid to remove residual catalyst and finally rinsed by deionized water and dried in an oven.

### Fabrication of CC-CNT@Fe_2_O_3_ and CC-CNT@NiO

**CC-CNT@Fe_2_O_3_:** Fe_2_O_3_ was coated on the CC-CNT by a simple aqueous reduction method. Firstly, the CC-CNT was immersed into FeCl_3_ (0.1 M) aqueous solution for 12 h, and then dried at 70 °C in drying oven for 2 h, and transferred into NaBH_4_ (0.1 M) aqueous solution for 6 h at room temperature afterwards. Finally, the obtained samples were washed by deionized water and dried in a drying oven for 12 h. **CC-CNT@NiO:** The NiO was coated on the CC-CNT by the same process with NiCl_2_ (0.1 M) aqueous solution instead of FeCl_3_ (0.1 M).

### Materials characterization

The morphologies and microstructures of the as-prepared samples were characterized by using a field-emission scanning electron microscope (FE-SEM, Hitachi S-4800) and a transmission electron microscope (TEM, FEI Tecnai F30) coupled with an energy dispersive X-ray spectrometer. Crystal structures were tested by X-ray diffraction (XRD, Philips, X’pert Pro, Cu Kα, 0.154056 nm). The vibrational information of chemical bonds of samples was characterized by micro-Raman spectroscopy (JY-HR 800, 532 nm wavelength YAG laser). The element composition and chemical bonding of samples were examined by X-ray photoelectron spectroscopy (XPS, PHI-5702, Mg KR X-ray, 1253.6 eV). The pore size distribution was measured by the Barrett–Joyner–Halenda method (ASAP 2020).

### Electrochemical characterization

The half-cell electrochemical properties were tested in a standard three-electrode system at room temperature on an electrochemical work station (CHI 600E, CH Instruments, Inc., China). 2 M KOH aqueous solution was used as the electrolyte, a Pt plate as the counter electrode, a saturated calomel electrode (SCE) as the reference electrode, and the CC-CNT@Fe_2_O_3_ or CC-CNT@NiO as the binder-free working electrode. Electrochemical impedance spectroscopy (EIS) curves were measured in frequencies from 100 kHz to 0.01 Hz at open-circuit voltage with an AC voltage perturbation amplitude of 5 mV. The mass of the active material was weighed by a microbalance (Mettler, XS105DU) with a tolerance of less than 0.01 mg.

## Supporting Information

File 1 Specific capacitance calculation and additional figures.

File 2 Video showing the ASC powering a blue LED for about 27 seconds.
