# Supplementary material for: Facile synthesis of carbon nanotube-supported NiO//Fe2O3 for all-solid-state supercapacitors
Source: Beilstein J Nanotechnol. 2019 Sep 23;10:1923–32. doi: 10.3762/bjnano.10.188 (PMC6774069; doi:10.3762/bjnano.10.188)
Supplement: File 1 — Specific capacitance calculation and additional figures. [file Beilstein_J_Nanotechnol-10-1923-s001.pdf]

BEILSTEIN JOURNAL OF NANOTECHNOLOGY

Supporting Information

for

Facile synthesis of carbon nanotube-supported NiO//Fe_2O_3 for all-solid-state supercapacitor*s*

Shengming Zhang, Xuhui Wang, Yan Li, Xuemei Mu, Yaxiong Zhang, Jingwei Du, Guo Liu, Xiaohui Hua, Yingzhuo Sheng, Erqing Xie and Zhenxing Zhang

Beilstein J. Nanotechnol. **2019,** *10,* 1923–1932. doi:10.3762/bjnano.10.188

Specific capacitance calculation and additional figures

License and Terms: This is a supporting information file under the terms of the Creative Commons Attribution License (http://creativecommons.org/licenses/by/4.0). Please note that the reuse, redistribution and reproduction in particular requires that the authors and source are credited.
The license is subject to the *Beilstein Journal of Nanotechnology* terms and conditions: (https://www.beilstein-journals.org/bjnano)

Calculation

The specific capacitance from GCD can be calculated [1] by the equation

$$c_s = \frac{I * \triangle t}{m * \triangle V} \quad \text{(S1)}$$

Where c_s is the specific capacitance (F g^{-1}), I is the response current (A), m is the mass of active material (mg), $\triangle t$ is the discharge time (s), and $\triangle V$ is the voltage window (V).

The capacity (*C*) of the single cell is calculated by the equation

$$C = \frac{c_s}{3.6} \quad \text{(S2)}$$

The specific capacitance of full cells can be calculated by equation (S1). The energy density of the device can be calculated by the equation

$$E = \frac{1}{2} * Cs * V^2 \quad \text{(S3)}$$

and the power density is given by

$$P = \frac{E}{t} \quad \text{(S4)}$$

Where E is the energy density (Wh kg^{-1}), Cs is the specific capacitance (F g^{-1}) calculated by GCD curves of device, V is the voltage window (V), P is the power density (W kg^{-1}), and t is the discharge time (s).

To maintain a charge balance between the cathode and anode, the mass ratio between the cathode (m_+) and anode (m_-) electrodes needs to follow the equation (S5):

$$\frac{m_+}{m_-} = \frac{C_- * \Delta V_-}{C_+ * \Delta V_+} \quad \text{(S5)}$$

Supporting Figures

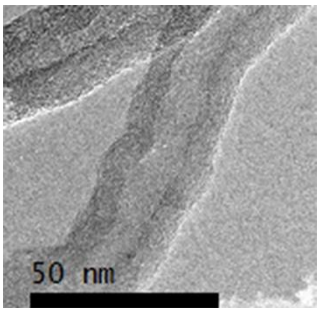

Figure S1: TEM of the CNT.

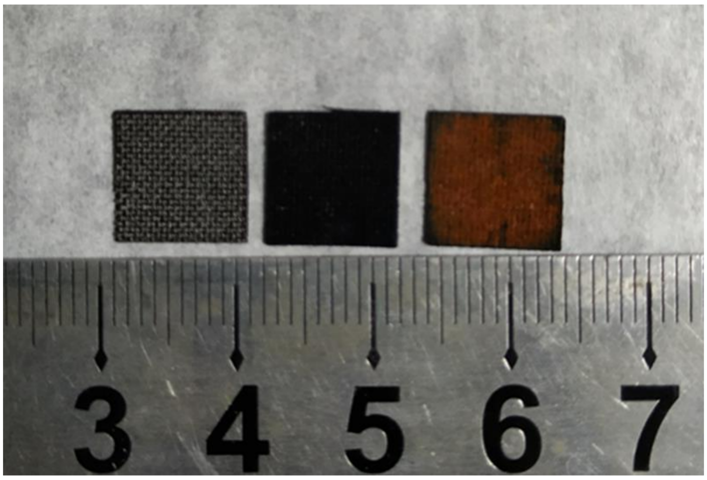

Figure S2: Optical photograph of CC, CC-CNT and CC-CNT@Fe_2O_3 (from left to right).

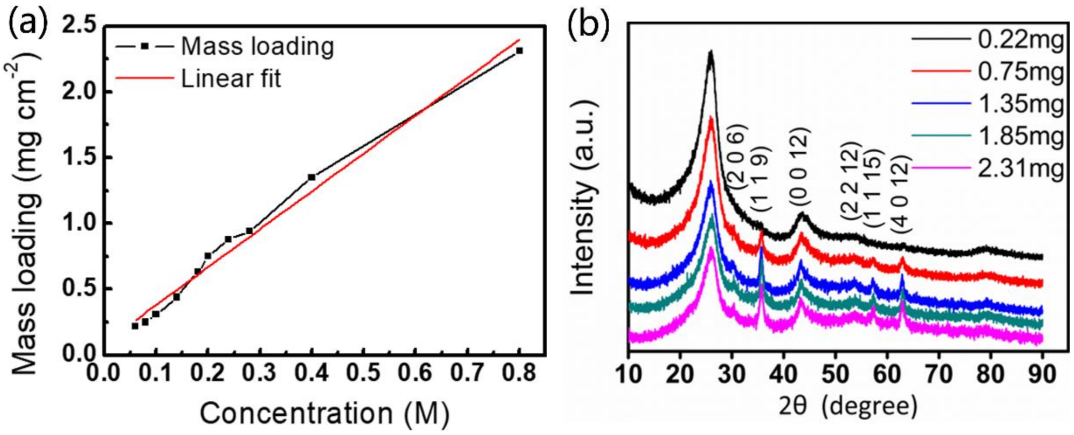

Figure S3: (a) Linear relationship between the mass loading of Fe_2O_3 on CC-CNT and the concentration of the $FeCl_3$ solution. (b) XRD of CC-CNT@Fe_2O_3 with different mass loadings of Fe_2O_3.

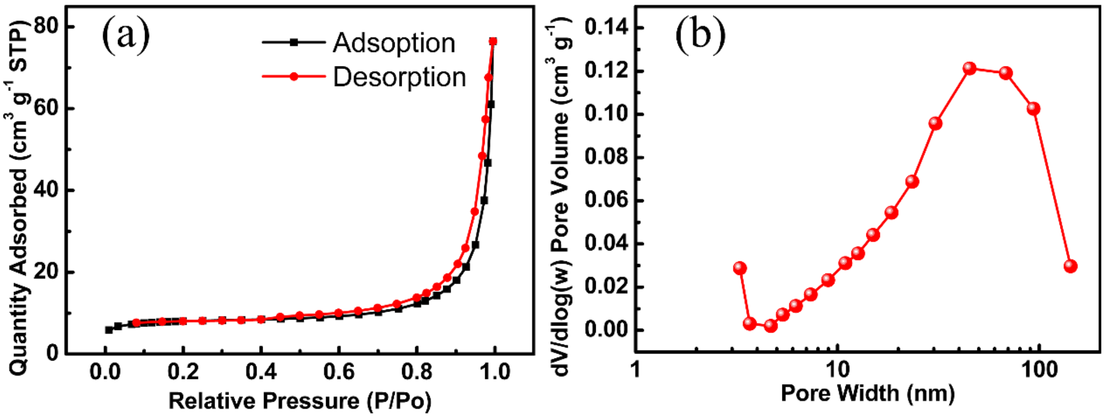

Figure S4: (a) N_2 adsorption-desorption isotherms and (b) BJH pore size distribution of CC-CNT@Fe_2O_3 at 75.9 K and (b) CC-CNT@NiO.

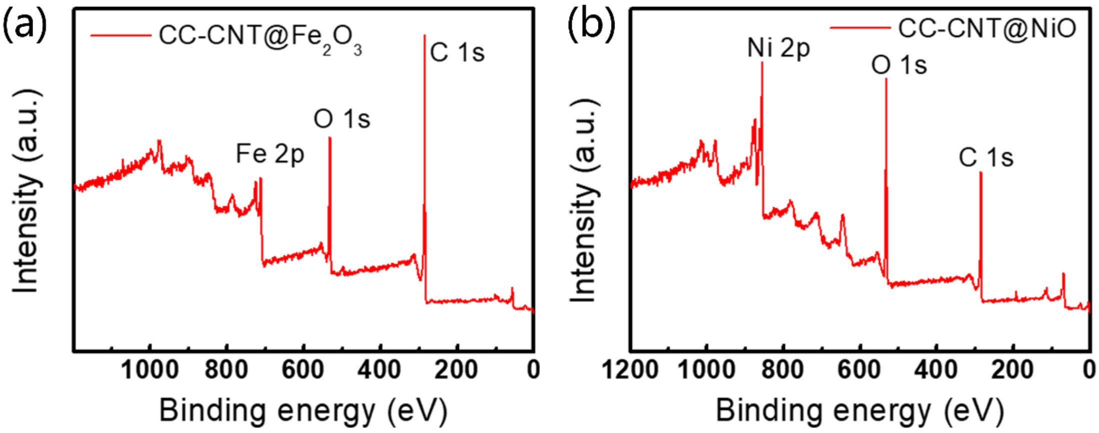

Figure S5: Whole XPS spectra of (a) CC-CNT@Fe_2O_3 and (b) CC-CNT@NiO.

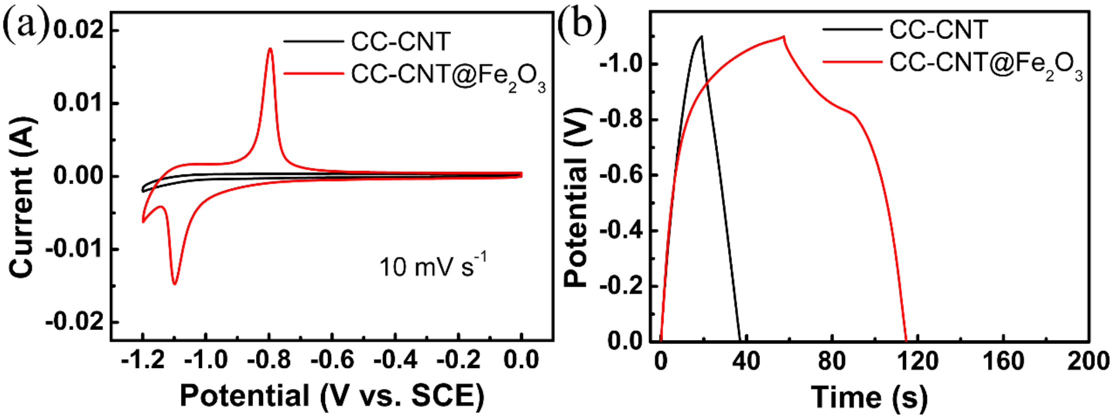

Figure S6: Comparison of CC-CNT and CC-CNT@Fe_2O_3: (a) CV curves at 10 mV s^{-1}, (b) GCD curves at 4.3 mA cm^{-2}.

Table S1: Fitted EIS values of CC-CNT@Fe_2O_3 and CC-CNT@NiO.

	Rs	CPE-T	CPE-P	Rct	W-R	W-T	W-P	C
CC-CNT @Fe_2O_3	1.125	0.0035572	0.85	0.002	0.35	0.056	0.37	0.8
CC-CNT @NiO	1.27	0.0012409	0.85	0.1	0.7	0.12	0.3327	0.465

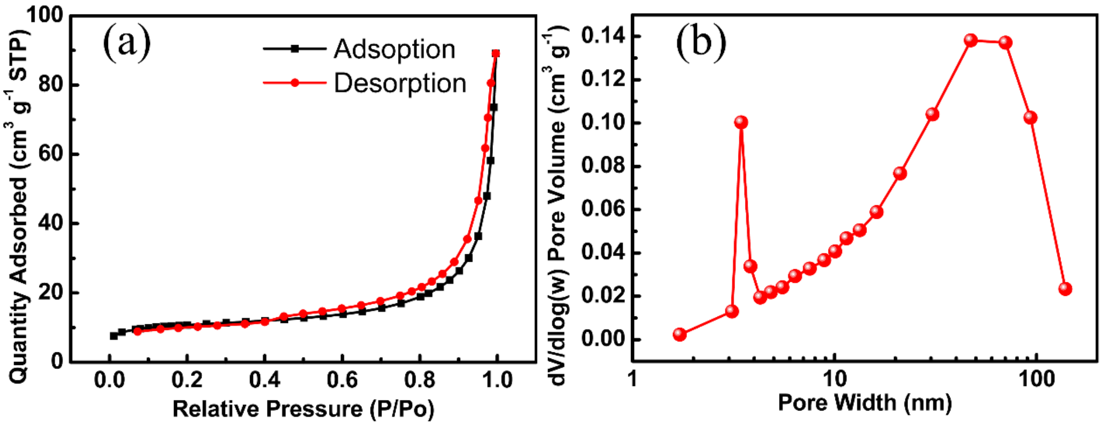

Figure S7: (a) N_2 adsorption-desorption isotherms and (b) BJH pore size distribution of CC-CNT@NiO at 75.9 K.

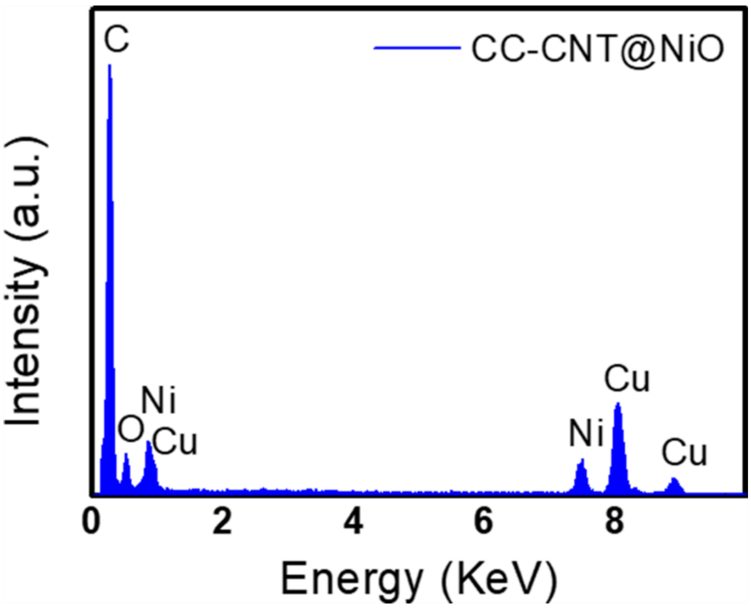

Figure S8: EDX of CC-CNT@NiO.

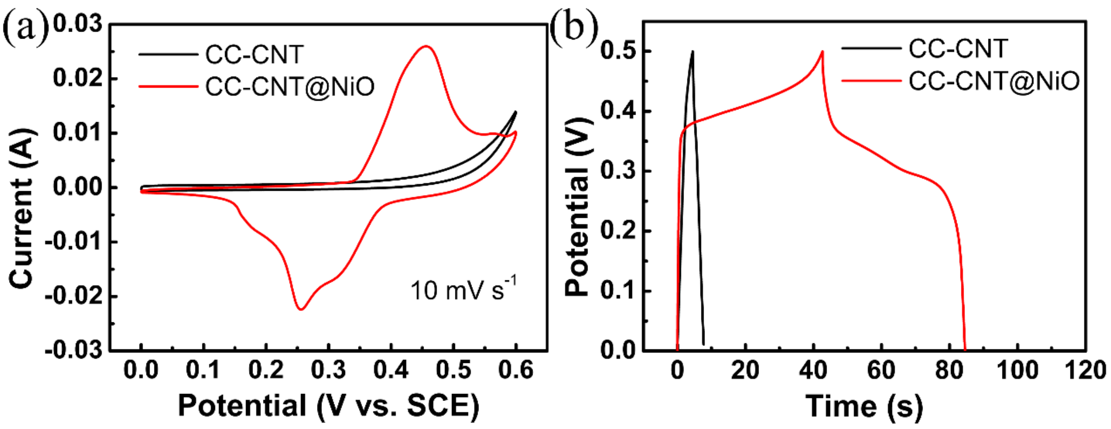

Figure S9: Comparison of CC-CNT and CC-CNT@NiO: (a) CV curves at 10 mV s^{-1}, (b) GCD curves at 6.6 mA cm^{-2}.

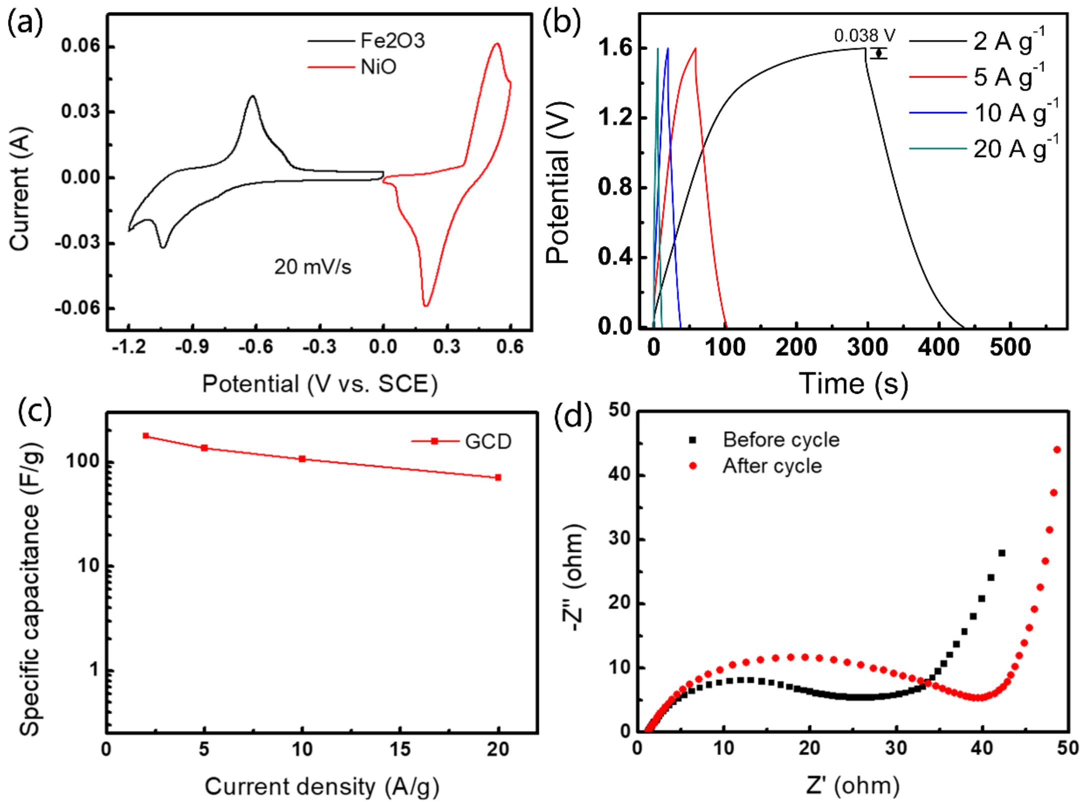

Figure S10: (a) CV curves of CC-CNT@Fe_2O_3 and CC-CNT@NiO at 20 mV s^{-1}. (b) GCD curves and (c) Specific capacitances at 2, 5, 10 and 20 A g^{-1} of the all-solid-state ASC. (d) EIS curve of the device before and after 5000 cycles.

References

[1] Zhou, J.; Zhao, H.; Mu, X.; Chen, J.; Zhang, P.; Wang, Y.; He, Y.; Zhang, Z.; Pan, X.; Xie, E. *Nanoscale* **2015,** *35*, 14697–14706. doi:10.1039/c5nr03426d
